# Determinants of sickness absence rate among Finnish municipal employees

**DOI:** 10.1080/02813432.2019.1568710

**Published:** 2019-01-28

**Authors:** Tiina Vuorio, Sakari Suominen, Hannu Kautiainen, Päivi Korhonen

**Affiliations:** a Department of Family Medicine, Institute of Clinical Medicine, University of Turku and Turku University Hospital, Turku, Finland;; b Department of Public Health, University of Turku, Turku, Finland;; c School of Health and Education, University of Skövde, Skövde, Sweden;; d Folkhälsan Research Center, Helsinki, Finland;; e Unit of Primary Health Care, Kuopio University Hospital, Kuopio, Finland;; f Health Center of Harjavalta, Central Satakunta Health Federation of Municipalities, Harjavalta, Finland

**Keywords:** Sickness absence, chronic disease, occupational health care, work ability, sociodemographic factor, health behavior, work-related factor

## Abstract

**Objective:** In addition to acute health problems, various aspects of health behavior, work-related and sociodemographic factors have been shown to influence the rate of sickness absence. The aim of this study was to concomitantly examine factors known to have an association with absenteeism. We hypothesized the prevalence of chronic diseases being the most important factor associated with sickness absence.

**Design:** A cross-sectional study.

**Setting:** Occupational health care in the region of Pori, Finland.

**Subjects:** 671 municipal employees (89% females) with a mean age of 49 (SD 10) years. Information about the study subjects was gathered from medical records, by physical examination and questionnaires containing information about physical and mental health, health behavior, work-related and sociodemographic factors. The number of sickness absence days was obtained from the records of the city of Pori.

**Main outcome measures:** The relationship of absenteeism rate with sociodemographic, health- and work-related risk factors.

**Results:** In the multivariate analysis, the mean number of chronic diseases (IRR 1.24, 95% CI 1.13 to 1.36), work ability (IRR 0.83, 95% CI 0.76 to 0.91), and length of years in education (IRR 0.90, 95% CI 0.85 to 0.95) remained as independent factors associated with absenteeism.

**Conclusion:** According to our results, chronic diseases, self-perceived work ability and length of years in education are the most important determinants of the rate of sickness absence. This implies that among working-aged people the treatment of chronic medical conditions is also worth prioritizing, not only to prevent complications, but also to avoid sickness absences.

**KEY POINTS**
- Various sociodemographic, health- and work- related risk factors have been shown to influence sickness absence.
- The study aimed to find the most important determinants of absenteeism among several known risk factors in Finnish municipal employees.
- Chronic diseases, self-perceived work ability and education years remained as the most important determinants of sickness absence rates.
- Treatment of chronic medical conditions should be prioritized in order to reduce sickness absence rate.

## Introduction

Writing certificates for sickness absence is seldom an easy task for a physician. In addition to the health of the employee, several additional factors have been shown to influence the rate and length of absenteeism. As markers of absenteeism the following have been identified: older age [1], female gender [2,3], lower grade of employment [3,4], alcohol consumption [5], smoking and obesity [6]**,** poor quality of sleep and low grade of leisure time physical activity [7]. Moreover, work-related stress [1,8] and low work engagement [9] seem to be independent risk factors of absenteeism. However, in the studies mentioned, the chronic illnesses of the employees have been studied with survey methodology or by using various measures on self-rated health.

To our knowledge, there are only two previous studies, in which chronic diseases have been verified as objectively as possible in population studies. Unfortunately even these fail to take into account work-related factors. Casimirri et al. demonstrated in a cross-sectional study of 514 sick-listed and non-sick-listed Italian workers an association between sickness absence days and the presence of two or more chronic diseases. Unfortunately, their evaluation did not include concomitant measurements of work-related factors [10].

Ubalde-Lopez et al. found that occurrence of multiple chronic health conditions was substantially associated with sickness absenteeism episodes due to cardiovascular, psychiatric or musculoskeletal diseases in a large (*n* = 372 370) sample of a Spanish working-age population . The effect of multi-morbidity was strongest among workers with no prior sickness absence. While the study was register-based, a variety of sociodemographic and work-related factors known to exert an influence on absenteeism were not taken into account [11].

The aim of this study was to concomitantly examine various factors known to be associated with sickness absenteeism. Since the number of sickness absenteeism days has been shown to predict morbidity and mortality [2,3] we hypothesized that the prevalence of chronic diseases is the most important factor associated with the rate of sickness absence.

## Material and methods

### Participants

The study is a part of the PORTAAT-study (PORi To Aid Against Threats), which is a longitudinal cohort study conducted among municipal employees of the city of Pori between 2014 and 2015 (39179 people aged 18–64 years were in the labor market in 2015) [12]. The PORTAAT-study aims to identify protective and risk factors for mental and cardiovascular health among working-aged individuals.

Invitation and information letters were sent to 2570 employees as an email attachment by the managers of the work units. The occupations of the invited employees represented a variety of professions like librarians, museum employees, groundkeepers, computer workers, social workers, nurses, physicians, administrative officials, and general office staff.

Altogether 836 employees (104/369 males, 732/2201 females) participated in the PORTAAT- study in 2014 yielding a participation rate of 33%. For the present analyses, we report data from the 671 participants who completed the follow-up visit in 2015.

### Physical examination

Physical examination was performed by trained study nurses. Height and weight were measured with the participant standing without shoes and outer garments. Weight was measured to the nearest 0.1 kg with calibrated scales and height to the nearest 0.5 cm with a wall-mounted stadiometer. Body mass index (BMI) was calculated as weight (kg) divided by the square of height (m^2^).

### Health-related factors

The Major Depression Inventory (MDI) [13] was used to evaluate depressive symptoms. Health-related quality of life was assessed with the EQ-5D questionnaire [14].

### Absenteeism records

The number of sickness absence days during the two-year time period from 1.1.2014 to 31.12.2015 was obtained from the records of the city of Pori. Absence due to taking care of a sick child at home was not included in the data. The total number of the sickness absence days during the two years’ time was divided by two to gain the average number of sickness absence days per year. There were 149 persons with no sickness absence days. For descriptive analysis, the rest of the study population was divided into tertiles according to the mean sickness absence days per year.

### Sociodemographic factors

Self-administrated questionnaires were used to gather information about the length of education in years and marital status. Subjective financial satisfaction was assessed with the question “I have to spare expenditures” (yes or no).

### Health behavior

Quality of sleep was assessed with a single question (good or not). Non-smoking was defined as having never smoked or having stopped smoking >12 months ago.

The 3-item Alcohol Use Disorders Identification Test (AUDIT-C) [15] was used in the evaluation of alcohol consumption. The cut-off point for harmful drinking was 5 points. Physical activity was assessed using a questionnaire about the frequency, intensity and duration of all weekly leisure time physical activity (LTPA) lasting longer than ten minutes. LTPA was categorized as follows:

- high: engaging ≥75 minutes per week of vigorous intensity activities or ≥150 minutes per week of moderate intensity activities or a combination of moderate and vigorous intensity activities.
- moderate: 0–149 minutes per week of moderate or vigorous intensity activities.
- low: no reported activity in moderate or vigorous intensity activities [16].

### Work-related factors

Work engagement was evaluated with the Utrecht Work Engagement Index UWES-9. UWES-9 consists of three subscales, vigor, dedication and absorption, which are scored on a 7-point Likert scale ranging from 0 (never) to 6 (daily) [17]. The Finnish values for total work engagement are <1.44 (very low), 1.44–3.43 (low), 3.44–4.53 (moderate), 4.54–5.30 (high) and 5.31–6.00 (very high) [18]. Work ability was assessed with the question: “What is your current work ability compared to your lifetime best?” This first item of the widely used Work Ability Index (WAI) [19] is named the Work Ability Score (WAS) and has a 0–10 response scale, where 0 represents “completely unable to work” and 10 “work ability at its best”. Reference values for WAS are suggested as for WAI; poor (0–5 points), moderate (6–7), good (8–9), excellent (10). WAS has a strong association with WAI and is trustworthy in evaluating work ability [20,21]. Physical workload was evaluated with a question and a 10 cm long visual analog scale (VAS) with advice for use: “How strenuous is your work physically? The mental work load was assessed with the question “How strenuous is your work mentally?” Answers were given with a 0–10 response scale (0 = very light to 10 = very hard). Bergen Burnout Indicator (BBI-15) was used to examine work stress. BBI-15 measures occupational burnout using 15 questions. The answers are given by using Likert-type scales from 1 to 6 (1 = completely disagree to 6 = completely agree), that are added together to form a score from 15 to 90, with the high score indicating high levels of work stress [22].

### Chronic diseases and regular medications

The information about chronic diseases and regular medications was gathered from medical records and self-administrated questionnaires. For detailed analysis, the diseases were categorized into eight different groups (musculoskeletal, cardiovascular, respiratory, gastrointestinal, neurological, mental, neoplastic, and diabetes mellitus).

### Informed consent and ethic approval

Study protocol and consent forms were reviewed and approved by the ethics committee of the Hospital District of Southwest Finland. All participants provided written informed consent for the project and subsequent medical research.

### Statistical analysis

Statistical significances for the unadjusted hypothesis of linearity across categories of the rate of sickness absence were evaluated by using the Cochran-Armitage test for trend and analysis of variance with an appropriate contrast (orthogonal). A multivariable forward stepwise Poisson regression model was used to determine the independent effects of sickness absenteeism. Variables significant at the *p* < .10 level in unadjusted analyses were included in the model. The Poisson regression model was tested using goodness-of-fit test of the model and the assumptions of overdispersion in the Poisson model was tested using the Lagrange multiplier test. All analyses were performed using STATA software, version 15.0 (StataCorp LP, College Station, TX).

## Results

The study population consisted of 671 municipal employees (89% females) with a mean age of 49 (SD 10) years. Table 1 displays a general overview of the study population and the characteristics of the study participants according to the mean annual rate of sickness absence days during the two-year follow-up. Twenty-two percent (149/671) [22.2% (95%CI: 19.1 to 25.5)] of the employees had no sickness absence days. The rest of the study population was divided into tertiles for descriptive analysis shown in the table. The mean incidence of sickness absence days per year during the study period was 11 days.

**Table 1.** Characteristics of the subjects according to the rate of sickness absence per year. Information about chronic diseases and absenteeism were register-based and the other variables self-reported.

	All *n* = 671	No absenteeism	1–8 days absent	9–26 days absent	Over 26 days absent	*p*-value for linearity
		*n* = 149	*n* = 178	*n* = 177	*n* = 167	
**Sociodemographic factors**						
Age, years, mean (SD)	49 (10)	47 (11)	49 (9)	49 (10)	50 (8)	.011
Women, n (%)	595 (89%)	130 (87%)	161 (90%)	154 (87%)	150 (90%)	.74
Education years, mean (SD)	13.7 (2.2)	14.0 (2.3)	14.2 (2.2)	13.5 (2.0)	13.1 (2.0)	<.001
Financial satisfaction, n (%)	490 (73%)	113 (76%)	141 (79%)	121 (68%)	115 (69%)	.036
Cohabiting, n (%)	545 (82%)	113 (67%)	152 (85%)	148(84%)	129(77%)	.72
**Health behavior**						
Smoking, n (%)	63 (9%)	12 (8%)	15 (8%)	9 (5%)	25 (15%)	.082
AUDIT-C, mean (SD)	2.9 (1.7)	3.1 (1.8)	2.8 (1.6)	2.9 (1.7)	2.9 (1.7)	.45
Good quality of sleep, n (%)	516 (77%)	119 (80%)	139 (78%)	140 (79%)	118 (71%)	.073
Leisure time physical activity, n (%)						.17
Low	135 (20%)	29 (19%)	36 (20%)	26 (15%)	44 (26%)	
Moderate	294 (44%)	61 (41%)	76 (43%)	90 (51%)	67 (40%)	
High	242 (36%)	59 (40%)	66 (37%)	61 (34%)	56 (34%)	
**Health-related factors**						
Number of chronic diseases, mean (SD)	1.2 (1.2)	0.8 (0.9)	1.0 (1.0)	1.2 (1.2)	1.8 (1.6)	<.001
Musculoskeletal	141 (21%)	13 (9)	28 (16)	39 (22)	61 (37)	<.001
Cardiovascular	134 (20%)	27 (18)	28 (16)	35 (20)	44 (26)	.036
Diabetes mellitus	26 (3.9%)	3 (2)	9 (5)	6 (3)	8 (5)	.36
Respiratory	51 (7.6%)	9 (6)	12 (7)	13 (7)	17 (10)	.16
Gastrointestinal	54 (8.0%)	5 (3)	14 (8)	11 (6)	24 (14)	<.001
Neurological	57 (8.5%)	7 (5)	13 (7)	17 (10)	20 (12)	.015
Psychiatric	28 (4.2%)	4 (3)	1 (1)	15 (8)	8 (5)	.035
Neoplastic	18 (2.7%)	1 (1)	3 (2)	7 (4)	7 (4)	.024
Number of regular medication, mean (SD)	1.1 (1.2)	0.6 (1.1)	0.8 (1.3)	1.2 (1.6)	1.7 (2.0)	<.001
Depressive symptoms (MDI), mean (SD)	5.0 (5.7)	4.4 (5.4)	4.2 (5.1)	5.7 (6.6)	5.7 (5.7)	.005
BMI, kg/m^2^, mean (SD)	26.8 (4.8)	26.4 (4.8)	26.2 (4.6)	27.0 (4.7)	27.7 (5.1)	.008
EQ-5D, score, mean (SD)	0.9 (0.1)	0.9 (0.2)	0.9 (0.1)	0.9 (0.1)	0.8 (0.2)	<.001
EQ-vas, mm, mean (SD)	82 (13.6)	86 (12)	85 (11)	82 (13)	76 (16)	<.001
**Work-related factors**						
Work engagement, UWES-9 score mean (SD)	4.8 (0.9)	4.9 (1.0)	4.9 (0.8)	4.7 (1.0)	4.7 (0.9)	.014
Work ability score, mean (SD)	8.3 (1.2)	8.7 (1.1)	8.6 (1.0)	8.2((1.2)	7.8 (1.6)	<.001
Physical workload, mm, mean (SD)	29.0 (26.0)	29 (27)	22 (23)	32 (27)	33 (27)	.019
Work stress (BBI-15), mean (SD)	32 (11)	30 (11)	31 (10)	32 (10)	33 (11)	.040
Mental workload, mm, mean (SD)	59.8 (21.7)	60 (22)	58 (22)	59 (22)	62 (21)	.34
Daytime work, n (%)	483 (72%)	103 (69)	148 (83)	135 (76)	114 (68)	.43

AUDIT-C: The Alcohol Use Disorders Identification Test for Consumption; MDI: Major Depression Inventory; EQ-5D: Quality of life; UWES-9: Utrecht Work Engagement; BBI-15: Bergen Burnout Indicator.

The participants with more absenteeism days were more likely to be older, less educated, and less satisfied with their financial situation. They also were more likely to be smokers, had more chronic diseases and regular medications, lower health-related quality of life, more depressive symptoms, and a higher BMI. Their work ability and work engagement were lower, they considered their work to be more strenuous physically and perceived more stress at work than employees with less sickness absence days.

When the variables with *P*-value <.10 in the univariate analysis (Table 1) were entered into the multivariate stepwise Poisson regression model, only the mean number of chronic diseases (IRR 1.24, 95% CI 1.13 to 1.36), work ability score (IRR 0.83, 95% CI 0.76 to 0.91), and education years (IRR 0.90, 95% CI 0.85 to 0.95) remained as independent factors associated with sickness absenteeism.

**Figure 1.**
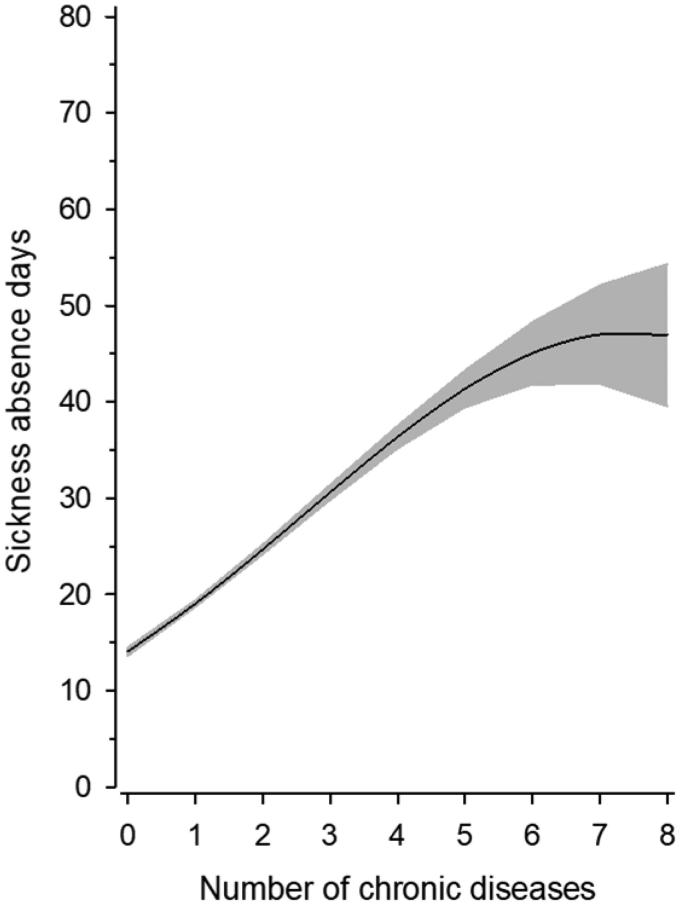
Relationship between number of chronic diseases and sickness absence days. The Poisson model was including quadratic terms for the number of chronic diseases and adjusted for age, gender, years for education and work ability score.

Figure 1 shows the adjusted relationship between sickness absence days and the number of chronic diseases. The rate of sickness absence days increased linearly with the number of chronic diseases.

## Discussion

According to the study, the presence of chronic diseases, the length of years in education and the self-perceived work ability were the most important determinants of the rate of sickness absence among Finnish municipal employees. The rate of absence days increased linearly with the number of chronic diseases.

Our results are in line with the findings of Casimirri et al. [10] and Ubalde-Lopez et al. [11], who also verified the association between multiple chronic conditions and the number of sickness absence days.

Our study is also in concordance with Sundstrup et al. [23], who recently in a sample of Danish general working-age population showed, that chronic diseases were associated with the risk of long-term sickness absences. In this study, the number of chronic diseases was self-reported and included eight specific disease categories. Of these, depression, malignancies and back disorders were significantly associated with the outcome. In our study, all disease categories except diabetes and respiratory diseases were associated with sickness absence.

In addition, Aaviksoo et al. reported that the most important factors associated with sickness absence in an Estonian sample of the working-age population were the presence of a chronic illness, poor self-rated health, low education and job dissatisfaction. In their study, all the variables in the analysis were dichotomized [24].

Of the numerous work-related factors evaluated in our study, self-perceived work ability appeared to be one of the most significant factors affecting the rate of absenteeism. Impaired work ability has been shown to be associated with chronic diseases, sickness absence and early retirement [20,25,26]. The occurrence of impaired work ability has been shown to increase with the number of concurrent chronic conditions, i.e. multimorbidity [23].

In our study, higher education was associated with a lower absenteeism rate, which is in concordance with other previous studies [3,4,24]. Lower educational status is often connected to more physically strenuous work, which in turn is also known to be a risk factor for absenteeism and decreased work ability [26].

In many previous studies, women seem to have more sick leaves than men [2,3]. However, in our study gender was not associated with absenteeism. We speculate that the reason for this difference in comparison to earlier studies is based on our study population which had a strong female dominance (89%).

### Strengths and limitations

The strengths of our study are that a wide range of data concerning somatic and mental health, sociodemographic, clinical and work-related factors were concomitantly taken into account. Absence data were obtained from the employer’s registry. Medical information concerning chronic diseases and medications were collected both from questionnaires and medical records in order to improve the accuracy of the data.

The major limitation of the study is its cross-sectional nature, which prevents any evaluation of causality of absenteeism with all the health- and work-related and lifestyle factors studied. The association between absenteeism and chronic diseases may be bidirectional. In some cases sickness absence could cause an increase in the health problem, rather than being a result of it.

A lot of the data used in the analysis was gathered with self-reported questionnaires resulting in the problem of the dependency within questionnaire data. Even if a wide range of different kinds of factors were evaluated in the study, there are still numerous factors, which can affect the level of absenteeism and cannot be assessed using self-reported questionnaires. The count of sickness absence days was obtained from the employer, so from an external source. Also information about chronic diseases and regular medication was completed from medical records. Unfortunately, we did not have the opportunity to gather more external data, which would have strengthened the external validity of the results. Self-reporting of factors examined might be unreliable, even though we tried to overcome this by using standardized procedures and validated questionnaires. Selection bias could have appeared, as individuals being absent from work at the time of the study were not responding. A possible healthy worker effect [27] might have affected the results, as individuals outside the workforce could not be included in the study. Unfortunately, we do not have more detailed characteristics of the non-respondents. The participation rate in the study was 33%. It is possible that some employees may have ignored the invitation to the study which was sent by e-mail. The reason for this might be the nature of the invitation to the study, which was by e-mail attachment. E-mail surveys generally seem to have about 20% lower response rate than mail surveys [28]. However, the mean age and sex ratio of the study participants were comparable to the entire personnel of the city of Pori [29]. Since the study population was female-dominated and all the participants worked in the public sector, a generalization of the results to all kinds of employees is not self-evident, on the other hand, it is hard to find arguments that would totally question any kind of generalization to the working age population in gainful employment. In Finland, the social service policies differ from other countries, especially from countries outside Northern Europe. Different social security systems might also have an impact on the sick leave rates in a specific country and hamper comparison of absenteeism studies between different countries.

Future studies might benefit from longitudinal design, collection of data from registries or other external sources, and other strategies for invitation of participants.

## Conclusion

The present study demonstrates how important it is to take even mild chronic diseases into account when assessing absenteeism and related factors. Also the employee’s own perception of his or her work ability asked with a simple, single-item question, seems to be a powerful tool for a physician. Because of the aging of the workforce, the number of employees suffering from multiple chronic conditions, I.e multimorbidity is likely to increase in the future [30]. Chronic conditions are too often ignored or adjusted in medical reports, which may give wrong impressions to general practitioners about the importance of the reasons for absenteeism. This implies that also among the working-age population the treatment and prevention of chronic medical conditions is worth prioritizing, not only in order to prevent complications or to decrease mortality but also in order to reduce sickness absence rate.
